# The promise of exosome applications in treating central nervous system diseases

**DOI:** 10.1111/cns.13743

**Published:** 2021-10-12

**Authors:** Jared Mattingly, Yuchen Li, Ji C. Bihl, Jinju Wang

**Affiliations:** ^1^ Department of Biomedical Sciences Joan C. Edwards School of Medicine Marshall University Huntington West Virginia USA; ^2^ Department of Pharmacology and Toxicology Boonshoft School of Medicine Wright State University Dayton Ohio USA

**Keywords:** central nervous system diseases, exosome‐based therapy, exosomes

## Abstract

Exosomes (EXs), a type of extracellular vesicles, are secreted from virtually all types of cells. EXs serve as cell‐to‐cell communicators by conveying proteins and nucleic acids with regulatory functions. Increasing evidence shows that EXs are implicated in the pathogenesis of central nervous system (CNS) diseases. Moreover, EXs have recently been highlighted as a new promising therapeutic strategy for in vivo delivery of nucleotides and drugs. Studies have revealed that infusion of EXs elicits beneficial effects on the CNS injury animal models. As compared to cell‐based therapy, EXs‐based therapy for CNS diseases has unique advantages, opening a new path for neurological medicine. In this review, we summarized the current state of knowledge of EXs, the roles and applications of EXs as a viable pathological biomarker, and EX‐based therapy for CNS diseases.

## Graphical abstract

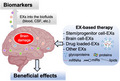

## 1 INTRODUCTION

Exosomes (EXs) are a major type of extracellular vesicles released by cells highly conserved from microorganisms to mammals.^1^ Their size ranges from 20 to 150 nm in diameter. About 50 years ago, they were initially considered cellular “waste.” In the early 1980s, EXs were recognized as distinct entities. Later in 1987, the term “exosome” was coined for these membrane particles. Over the past decades, increasing evidence shows that EXs can cross the blood‐brain barrier (BBB) and serve cell‐to‐cell communicators via conveying their carried biological cargoes (nuclear acids, proteins, and lipids).^2^, ^3^ In the central nervous system (CNS), EXs are released from virtually all brain cells, including neurons, astrocytes, microglia, and endothelial cells.,^4^, ^5^ and exchange molecule messages related to neuronal function and neurotransmission in the brain via the reciprocal communication between brain cells.

Given the capability of EXs to reach body compartments and connect origin cells with target cells, EXs have a promising potential to be used in clinical applications. Indeed, a large body of preclinical studies has shown the implication and therapeutic potential of EXs in CNS diseases. Here, we review the current state of the knowledge of EXs, the roles and applications of EXs as a viable pathological biomarker, and EX‐based therapy for CNS diseases.

## 2 OVERVIEW OF EXS

### 2.1 EX biogenesis, isolation, and contents

EX generation is initiated by a process that involves double invagination of the plasma membrane and the formation of intracellular multivesicular bodies (MVBs). The MVBs contain intraluminal vesicles that are secreted as EXs via exocytosis and MVB fusion to the plasma membrane.^6^, ^7^ Although the exact biogenesis mechanism has not been fully delineated, multiple molecules including Ras‐related protein GTPase Rab, tumor susceptibility gene 101 (TSG101), tetraspanins, apoptosis‐linked gene 2‐interacting protein X (ALIX), endosomal sorting complexes required for transport (ESCRT) complex, ceramides, etc., have been reported to be involved in the biogenesis of EXs.^6^, ^8^, ^9^

The methodologies for isolating EX include ultracentrifugation,^10^ size‐based isolation,^11^, ^12^, ^13^ microfluidic method,^14^, ^15^ and commercial kits such as Exo‐Spin, Total EX Isolation, and ExoQuick, although a gold‐standard method is still lacking. The choice of isolation methods depends on the experiment's purpose. Deun et al.^16^ compared different EX isolation methods and found that ultracentrifugation and OptiPrep density gradient centrifugation had purer EX preparations than ExoQuick and Total EX Isolation precipitation did, while another group reported that circulating EXs isolated by ExoQuick precipitation produced exosomal RNA and protein with higher purity and quantity than ultracentrifugation.^17^

The biological molecular cargos of EXs include proteins, lipids, and genetic materials. Exosomal proteins include constitutive proteins such as tetraspanins (CD9, CD63, etc.) that could be used to identify EXs and cell type‐specific proteins such as histocompatibility complex (MHC) class‐II on cells that express MHC class‐II.^18^ The lipid composition of EXs is rich in glycolipids and free fatty acids.^19^, ^20^ Exosomal genetic materials, including messenger RNA, microRNA (miR), and long noncoding RNA (lncRNA),^21^, ^22^, ^23^ have been demonstrated to play critical roles in pathophysiological processes since they are protected from degradation due to the double lipid membrane of EXs.^24^, ^25^

### 2.2 EX characterization and uptake routes

Transmission electron microscopy (TEM) is the most common methodology used for EX characterization. Under TEM, EXs display a cup‐shaped or spherical morphology and membrane‐enclosed structures. To analyze the quantity and size distribution of EXs, light scattering technologies including nanoparticle tracking analysis (NTA) are widely used. To specify the cellular origin of EXs, our group has developed a sensitive and specific method for purifying and phenotyping EXs from plasma by taking the advantage of EXs carrying parent cell antigens.^26^ We isolated endothelial cell‐derived EXs from plasma by using endothelial cell‐specific markers (CD144/CD105) and EPC‐EXs with EPC‐specific markers (CD34/KDR). Recently, Liu et al.^27^ identified keratinocyte extracellular vesicles with keratinocyte marker calcium‐sensing receptor from human skin biopsy tissue and plasma.

EX‐mediated communication process mainly includes release, transport, capture, internalization, and regulation.^28^, ^29^ Although whether EXs are selectively incorporated into recipient cells is still unknown yet, currently known cellular internalization routes include clathrin‐ or caveolin‐dependent endocytosis, phagocytosis, macropinocytosis, lipid raft‐mediated internalization, and membrane fusion.^30^, ^31^ Chemical inhibitors such as heparin,^6^ chlorpromazine,^32^ wortmannin,^33^ dynasore,^34^ and omeprazole^35^ have been applied to specify the uptake pathways of EXs. Due to their intrinsic properties, such as high biocompatibility and biodegradability, low toxicity and immunogenicity, and nanometric size,^6^, ^36^ EXs are promising drug vehicles. Therefore, future studies are required to identify the exact uptake mechanisms of EXs that will facilitate establishing an effective EX delivery system.

### 2.3 EX function varies upon the origin of cells and the cellular status

Abundant studies have documented that EXs can convey their carried cargoes to induce physiological changes in nearby or distant recipient cells. Of note, the functions of EXs vary based on the origin of cells and their cellular status. Obesity can affect the functions of adipose tissue macrophage‐derived EXs on modulating insulin sensitivity through altering EX carried miR‐155.^37^ Our group has demonstrated that EPC‐derived EXs (EPC‐EXs) released under starvation or inflammation stimulation function differently on hypoxia/reoxygenation‐injured endothelial cells, which might be related to their carried miR‐126.^38^ Under conditions of high neuronal activity and/or oxidative stress, glia has been shown to release synapsin via EXs to modulate glia‐neuron interaction.^39^ EXs isolated from cerebrospinal fluid (CSF) of traumatic brain injury patients contain several known traumatic brain injury biomarkers such as cytoskeletal proteins and neurite outgrowth‐related proteins.^40^ Recently, increasing studies show that exercise intervention can regulate EX release and function. Endurance exercise‐derived EXs have a great potential for treating metabolic disease^41^ and circulating EXs carrying cardioprotective miRs in exercised rats, although the origin of EXs is unknown.^42^ We revealed that exercise intervention can modulate the release and function of circulating EPC‐EXs by affecting the package of miR‐126 into EXs.^43^ Besides modifying the status of parent cells and exogenous stimulation as above discussed, researchers have developed several other engineering strategies including modification of isolated EXs and EX‐functionalized biomaterials to promote the use of EXs as nanomedicines. Sun et al.^44^ successfully incorporated the anti‐inflammatory agent, curcumin, into EXs. A recent report shows that engineered extracellular vesicles with synthetic lipids can achieve an effective gene silencing effect in vitro study.^45^ Jang group utilized macrophages and monocytes‐derived nanovesicles, as drug carriers to successfully load doxorubicin. They found that these drug‐loaded vesicles trafficked to tumor tissues, resulting in reduced tumor growth in mice bearing CT26‐tumor xenografts.^46^ Additionally, the use of biomaterials may provide a platform to enhance EX bioavailability, sustain release, and maximize EX regenerative capacity. Chen et al.^47^ investigated delivering EPC extracellular vesicles incorporated within a composite hyaluronic acid gel for treating myocardial infarction, which was found to enhance peri‐infarct angiogenesis in a rat model of myocardial infarction. Together, these findings suggest that EXs are engineerable and the engineered extracellular vesicles may also hold great potential for clinical applications.

## 3 THE ROLES OF EXS IN CNS INJURY DISEASES

### 3.1 Strokes

Stroke is the second leading cause of death globally. Ischemic stroke accounts for around 87% of all strokes,^48^ and the remainder is hemorrhagic stroke. The pathophysiology of stroke is complex and involves numerous interrelated and coordinated events, including peri‐infarct depolarization, excitotoxicity, oxidative stress, inflammatory response, and BBB disruption, which leads to the death of brain cells (neurons, endothelial cells, astrocytes, microglia, etc.). Stroke recovery is planned by a series of highly interactive processes involving angiogenesis, neurogenesis, synaptogenesis, and neuronal and synaptic plasticity.

Abundant studies have demonstrated that almost all brain cells can release EXs. In 2006, Faure et al.^5^ described the release of EXs by cortical neurons in vitro. They found that EXs released by cortical neurons and the exosomal release are regulated by depolarization, suggesting that EXs may have a regulatory function at synapses and could allow an intercellular exchange of signals within the brain. Indeed, increasing evidence indicates that brain cell‐derived EXs, such as astrocyte‐EXs and microglia‐EXs, are important components of the brain microenvironment and play important roles in brain injury progression and repair. Astrocyte‐EXs exhibit neuroprotective effects by alleviating inflammation/apoptosis and enhancing neuroplasticity,^49^ but also can induce neuroinflammation and neurotoxicity.^50^ Astrocyte‐EXs released under anti‐inflammatory cytokines such as interleukin‐10 stimulation contain a set of neurotrophic proteins that promote neurite outgrowth and neuronal plasticity/survival.^49^ In the contrast, astrocyte‐EXs released in response to inflammatory stimuli like interleukin‐1 beta and tumor necrosis factor‐alpha were enriched with miR‐125a‐5p and miR‐16‐5p that can target neurotrophic signaling proteins, NTKR3 and Bcl2, in neurons. The downregulation of these proteins resulted in decreased dendritic growth, reduced spike rates, and burst activity.^50^ Microglia are resident immune cells in the brain that are involved in regulating immune response and brain function. Microglia‐EXs have previously been shown to regulate synaptic transmission by promoting the neuronal production of ceramide and sphingosine to enhance excitatory neurotransmission.^51^, ^52^ More recently, increasing studies uncover the roles of microglial responses in neurovascular injury^53^, ^54^ and brain repair in experimental mouse stroke models.^55^, ^56^ Vexler and colleagues show that the microglia‐monocyte signaling contributes to cerebrovascular leakage and inflammation in a mouse model of childhood stroke.^54^ Notably, the pro‐inflammatory phenotype of microglia could be polarized toward a “healing” phenotype.^53^ While in aged mice, microglia demonstrated reduced interaction with neighboring neurons and diminished polarity toward the infarct lesion which may contribute to aging‐driven vulnerability and poorer recovery after ischemic stroke.^55^ It is possible that EXs, through miRs and proteins, may both regulate microglial responses and mediate microglia‐afforded biological effects in the brain. Future studies identifying the microglia EX‐carrying miRs could lead to new therapies for stroke.

Besides endogenous EXs mediate brain cell crosstalk, exogenous EXs also play a role in modulating the talking of brain cells. Human umbilical vein endothelial cells‐derived EXs could attenuate hypoxia/reoxygenation‐induced apoptosis in neurons by conveying miR‐21‐3p to inhibit the ATG12 signaling.^57^ Our group reported that EPC‐microvesicles rescued endothelial cells from ischemia‐induced apoptosis in vitro.^38^ Later on, we demonstrated that miR‐210 enrichment could boost the protective effects of EPC‐EXs on endothelial cells against mitochondrial dysfunction.^58^ Recently, another group shows that the EXs isolated from the plasma of cerebral ischemic preconditioned mice could rescue N2a cells against oxygen‐glucose deprivation injury, via conveying the exosomal miR‐451a to directly target the Rac1/PAK1 signal pathway in recipient neurons.^59^ Angiogenic miRs such as miR‐132‐3p‐modified MSC‐EXs have also been shown to protect endothelial cells against ischemic injury through regulating the redox balance and tight junction protein expression.^60^

Due to the role of EXs plays in the brain, researchers proposed that EXs might be a novel source for treating stroke which has been investigated in stroke animal models. Chen et al.^61^ found that intravenous administration of adipose‐MSCs derived EXs (AD‐MSC‐EXs) markedly reduced the infiltration of CD11+ and CD68+ cells, decreased oxidative stress, and increased angiogenesis in the rat after acute ischemic stroke. Adipose‐derived stem cells released EXs are also shown to alleviate acute cerebral stroke injury via interfering macrophage phenotype^62^ and promote functional recovery in the chronic phase.^63^ Besides, the M2 microglia‐EXs also exhibit a neuroprotective effect.^64^ In an in vitro, M2 microglia‐EXs could be internalized by neurons to attenuate oxygen‐glucose deprivation‐induced injury. In an MCAO‐induced stroke mouse model, infusion of M2 microglial‐EXs attenuated infarction area and improved behavioral deficits via targeting ubiquitin‐specific protease 14.^64^ MiRs play various roles in CNS injuries.^65^ For instance, miR‐98 protects against blood‐brain‐barrier breakdown and improves stroke outcomes.^66^ Increasing evidence indicates that exosomal miRs are one of the major executors of EXs in stroke. Indeed, Xin et al.^67^ found that miR‐17‐92 cluster‐enriched MSC‐EXs could increase neural plasticity and functional recovery of stroke in a rat model. They further revealed that these EXs can enhance axon‐myelin remodeling and motor electrophysiological recovery through activating the PI3K/Akt/mTOR pathway.^68^ Another study shows that infusion of miR‐132‐3p‐primed MSC‐EXs can reduce cerebral vascular oxidative, improve BBB function, and alleviate brain injury in a transient MCAO mouse model.^60^ MiR‐126 is involved in vascular regulation and vascular cognitive impairment.^69^ We recently have demonstrated that EXs from miR‐126‐modified EPCs can preserve cerebral blood flow on the acute phase, elevate angiogenesis/neurogenesis in the peri‐infarct area, and improve neurological function on day 14 in the type 2 diabetic stroke mouse model.^70^ Intriguingly, a recent study reported that a combination of cerebral endothelial cells‐extracellular vesicles and tissue plasminogen activator (tPA) can elicit neurovascular protection effect and promote neurological recovery in a rat model of embolic and transient middle cerebral artery occlusion.^71^ The authors observed that the therapeutic effects were ascribed to the extracellular vesicle carried miRs (miR‐328a, ‐483, ‐125b, etc.) which could reduce tPA‐ and ischemia‐increased proteins and thereby mediating thrombolysis and BBB integrity. These studies suggest the potential role of exosomal miRs in improving neurovascular function in stroke has the potential for clinical translation.

The changes of circulating exosomal contents in stroke are reported in clinical studies, suggesting the biomarker promising of EXs. Kerr et al.^72^ found that the levels of inflammasome protein caspase‐1 and apoptosis‐associated speck‐like proteins, caspase‐recruitment domain, and IL‐18 were upregulated in EXs collected from the serum of stroke patients. In patients with acute stroke, serum exosomal miR‐9 and miR‐124 levels were higher.^73^ What's more, the level of both miRs was positively correlated with the NIHSS score and infarct volumes, suggesting that serum exosomal miR‐9 and miR‐124 are promising biomarkers for diagnosing acute ischemic stroke and evaluating the degree of damage caused by ischemic injury. Of note, there is an increasing body of evidence that shows the differences of extracellular vesicles in males and females. For example, sex‐specific changes in brain‐derived extracellular vesicles biogenesis and protein cargo signatures have been identified in rats post nicotine self‐administration.^74^ The synovial fluid extracellular vesicle protein content is altered in a sex‐specific manner with osteoarthritis^75^ which explains the increased prevalence and severity of osteoarthritis in women. In stroke research, sex differences also have been found at cellular levels^76^ and the whole animal.^77^ The characteristics of activated microglia after stroke in males and females are shown to be different which may cause a difference in the pro‐ and anti‐ inflammatory responses after stroke.^76^ However, there is little research regarding sex differences in EXs and EX effects. Future research targeting sexual dimorphism in EXs may help to further the development of EX‐based therapy for stroke.

Although an abundance of preclinical evidence shows the promise of EXs on treating stroke, for clinical applications, further investigation is needed, such as in the mechanisms of interaction between EXs and target brain cells, circulation kinetics, biodistribution, and metabolism of EXs. There is an ongoing clinical trial (Identifiers# NCT03384433) sponsored by Isfahan University of Medical Sciences. It aims to evaluate the administration of allogeneic MSC‐EXs enriched with miR‐124 on the improvement of disability in patients with acute ischemic stroke. No relative results have been released so far.

### 3.2 Vascular dementia

Vascular dementia (VD) often appears concurrently with cerebral small vessel disease as sequelae to chronic hypertension.^78^ Myelinated brain tissue is most often affected in VD, along with calcification of arterioles being primary pathologic manifestations.^79^ VD is one of the most common causes of dementia after Alzheimer's and Parkinson's diseases; therefore, early diagnosis of VD is important for preventing the occurrence and development of dementia.

Some studies indicating that exosomal miRs are implicated in the pathogenesis of VD. Yang and colleagues found that exosomal miR‐135a level in the plasma was increased while miR‐193b level was decreased in patients with VD.^80^ The levels of some proteins such as glucose transporter 1(GLUT1), and large amino acid transporter 1 (LAT1) in plasma EXs were found to be significantly higher in subjects showcasing white matter hyperintensity in comparison to those without.^81^ In a recent study, Barbagallo et al. compared serum ex‐miR expression in samples from patients with VD and healthy controls. They found the levels of serum exosomal miR‐23a, miR‐29a, and miR‐130b were significantly decreased in VD patients.^82^ Collectively, these findings indicate the promising of using exosomal miRs as VD biomarkers, but there is limited study regarding EX‐based therapy for VD.

### 3.3 Alzheimer's disease

Alzheimer's disease (AD) is a late‐onset neurodegenerative disorder involving memory and decline in cognition, holding the title of the most prevalent cause of dementia. The pathogenic hallmarks of AD include the accumulation of amyloid and tau proteins^83^, ^84^ as well as neuroinflammation, which eventually results in damage and death of brain cells.^84^

Several studies have noted that EXs are involved in the pathogenesis of AD. As early as 2006, Simons et al.^85^ reported that Aβ peptides can be secreted from HeLa and N2a cells in association with EXs. Later on, increased levels of Aβ and tau were detected in serum EXs in a transgenic mouse model of AD.^86^ Mattson and colleagues reported that Aβ carried by extracellular vesicles isolated from the CSF and plasma of AD patients could impair mitochondrial function and sensitize neurons to excitotoxicity.^87^ In the clinic, Hallbeck et al.^88^ found that EXs from AD patients' brains contain an increased level of Aβ oligomers and these EXs can mediate neuron‐to‐neuron propagation of Aβ oligomers in a co‐culture system. Meanwhile, EXs could accelerate α‐synuclein aggregation. EXs secreted from activated microglia are indicated to be important mediators of α‐synuclein‐induced neurodegeneration in PD.^89^ PD‐linked human ATP13A2/(PARK9) can promote α‐synuclein externalization via EXs.^90^ Additionally, the biomarker role of EXs in AD is also highlighted. Researchers found that ex‐miR‐193b was significantly lower in AD patients and negatively correlated with Aβ42 expression, suggesting that EX‐miR‐193b could be used as a potential diagnostic biomarker to reflect the progression of AD.^91^ In clinical studies, Gui et al.^92^ identified that exosomal miRs such as miR‐1 and ex‐miR‐19b‐3p were decreased, while miR‐153, miR‐409‐3p, miR‐10a‐5p, and let‐7g‐3p were increased in the CSF of patients with PD. Cha et al.^93^ found that miR‐212 and miR‐132 were downregulated in neural‐EXs isolated from the blood of AD patients.

Besides being implicated in the pathogenesis and serving as biomarkers of AD, EXs may also elicit neural protective effects. Glatzel et al. found that prion protein (PrP^C^) was highly enriched on EXs isolated from both human and mouse neuroblastoma cell lines. They found these EXs exhibited a high binding affinity for dimeric, pentameric, and oligomeric Aβ species to accelerate fibrillization of amyloid‐beta, thereby reducing neurotoxic effects imparted by oligomeric Aβ.^94^ Another in vitro study shows that up‐regulation of EX secretion from SMS2 siRNA‐pretreated neurons enhances Aβ uptake into microglia and significantly decreases extracellular levels of Aβ in neurons, indicating a novel mechanism responsible for clearance of Aβ through EXs.^95^ Intranasal administration of EXs derived from cytokine preconditioned MSCs has been shown to activate microglia and increase dendritic spine density in the brain, leading to neuroprotection in a 3xTg model of AD.^96^ EXs were also designed as a carrier for the anti‐inflammatory agent curcumin to effectively deliver curcumin across the BBB, and thereby leveraging the AKT/GSK‐3β pathway to inhibit Tau phosphorylation and prevent neuronal death and thereby alleviating AD symptoms.^97^

### 3.4 Parkinson's disease

Parkinson's disease (PD) is the second most common neurodegenerative disease after AD around the world.^98^ The features of PD pathology include the progressive loss of dopaminergic neurons in the substantia nigra pars compacta and the formation of Lewy bodies, which mainly contain misfolded α‐synuclein.^98^ Researchers have proposed a hypothesis that PD may begin in the olfactory bulbs or enteric nervous system, afterward, spread to other brain regions during disease progression.^99^

Increasing studies suggest that EXs and their carried genetic materials are implicated in PD. Several exosomal miRs (miR‐27a‐3p, miR‐125a‐5p, etc.) collected from CSF are different in PD patients as compared to the healthy controls.^100^ The lnc‐MKRN2‐42:1 in plasma EXs positively correlated with the movement disorder score, suggesting exosomal lncRNAs are involved in the pathogenesis of PD.^101^ Santos et al.^102^ identified CSF exosomal miR biomarker panels including let‐7f‐5p, miR‐27a‐3p, miR‐125a‐5p, miR‐151a‐3p, and miR‐423‐5p for early diagnosis of PD. In another study, the investigators found that plasma exosomal miR‐331‐5p was significantly upregulated while the exosomal miR‐505 was downregulated in PD patients.^103^ More recently, Morris and colleagues revealed that Lewy body disorders extracellular vesicles constitute a “pathological package” that is capable of inducing aggregation of wild‐type alpha‐synuclein.^104^

Due to the non‐immunologic nature of EXs, they could also be used as a delivery vehicle for therapeutic molecules crossing the BBB.^105^ Indeed, a recent study reveals that dopamine‐loaded blood EXs increase the delivery of dopamine to striatum and substantia nigra >15‐fold than traditional methods.^106^ Kojima et al.^107^ found that catalase mRNA‐loaded EXs were capable of attenuating neurotoxicity and neuroinflammation in 6‐hydroxydopamine (6‐OHDA) or LPS‐induced mouse models of PD. In another study, the catalase‐loaded EXs elicited neuroprotective effects in in vitro PC12 neuronal cells and in vivo 6‐OHDA model of PD.^108^ In addition, EXs modified with brain‐specific rabies virus glycoprotein and DNA minicircles can downregulate BACE1/α‐synuclein in PD.^109^ Collectively, although there are no therapies currently available to halt the progression of PD, EXs hold great potential as a delivery vehicle for treating PD.

## 4 CONCLUSION AND PERSPECTIVES

**FIGURE 1:**
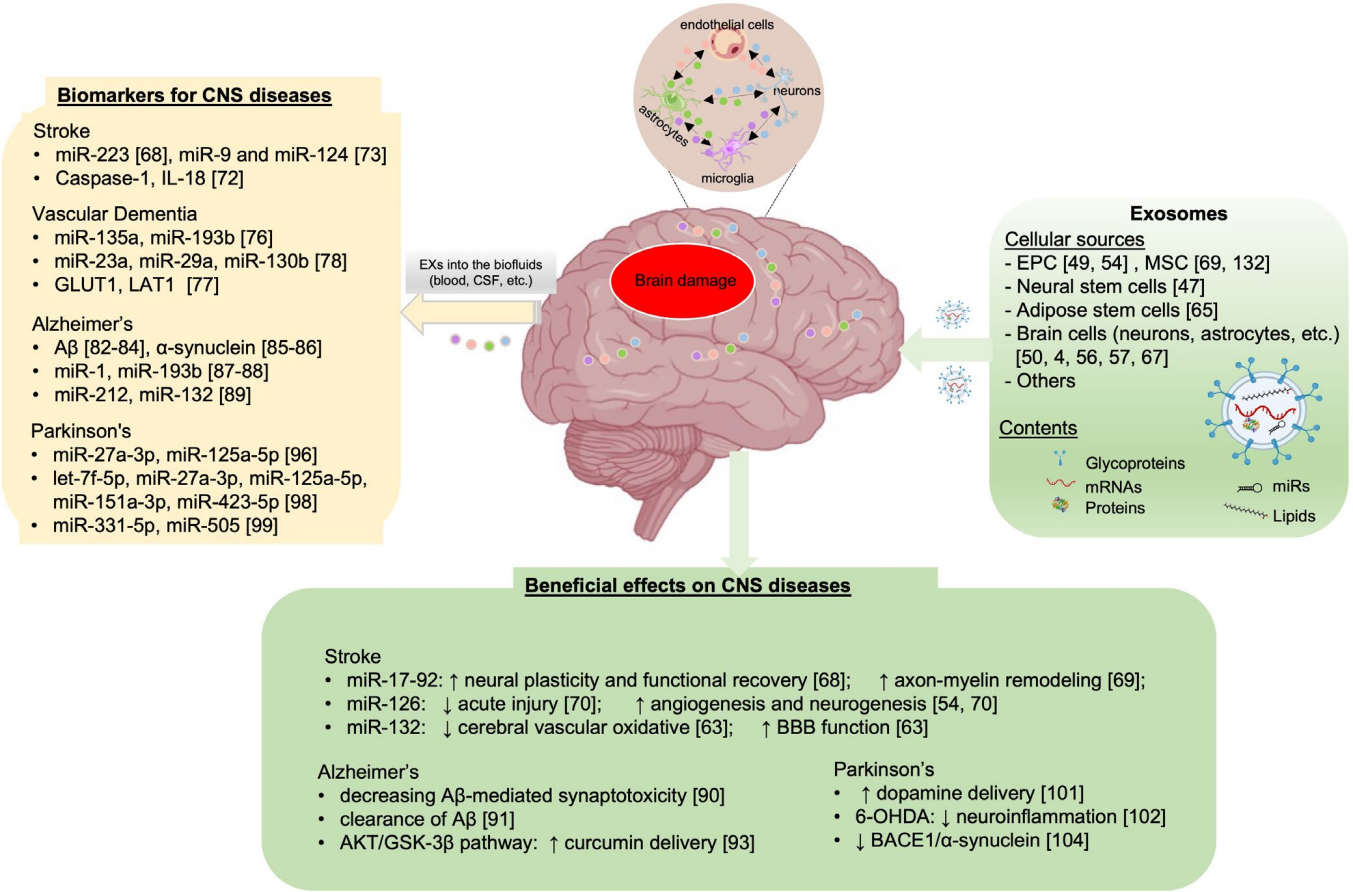
Roles of EXs in CNS diseases. EXs could be released from various types of cells and contain different cargoes such as miRs, lncRNAs, and proteins. EXs have a great promise of being used as biomarkers for diagnosis and prediction of CNS diseases, or as a cell‐free therapy for treating CNS diseases. Although the underlying mechanisms responsible for the exosomal beneficial effects have not been fully understood, it might be partially related to the capacity of EXs to pass through the BBB and to convey genetic materials (miRs, lncRNAs, etc.) and/or proteins to modify gene and protein expressions in the target cells and tissues, ultimately leading to reduced neuroinflammation and improved angiogenesis and neurogenesis in the brain

In conclusion, EXs play a physiological role and are implicated in the pathogenesis of CNS diseases including stroke, VD, PD, and AD. Administration of EXs has been shown to elicit neuroprotective and neurorestorative effects in rodent CNS models, indicating a great promising for treating CNS diseases. Compared with cell‐based therapy, cell‐free EX‐based therapy has the advantage of simple preservation/transfer, modification potential, can cross the BBB, low immunogenicity, inability to proliferate, lack of risk of cellular injection‐induced vascular occlusion, etc.^110^ In addition, as a drug delivery vehicle, EXs have the advantages of prolonging drug half‐time, maximizing biological compatibility, minimizing systemic toxicity, and penetrating deep tissues. Concurrently, targeting specific receptors using EX‐mediated binding to transferrin receptors, low‐density lipoprotein receptors, and insulin receptors is under development, which will enhance the promising of EXs for treating CNS diseases. The potential clinical biomarker application and therapeutic effects of EXs on CNS diseases are summarized in Figure 1.

**FIGURE 2:**
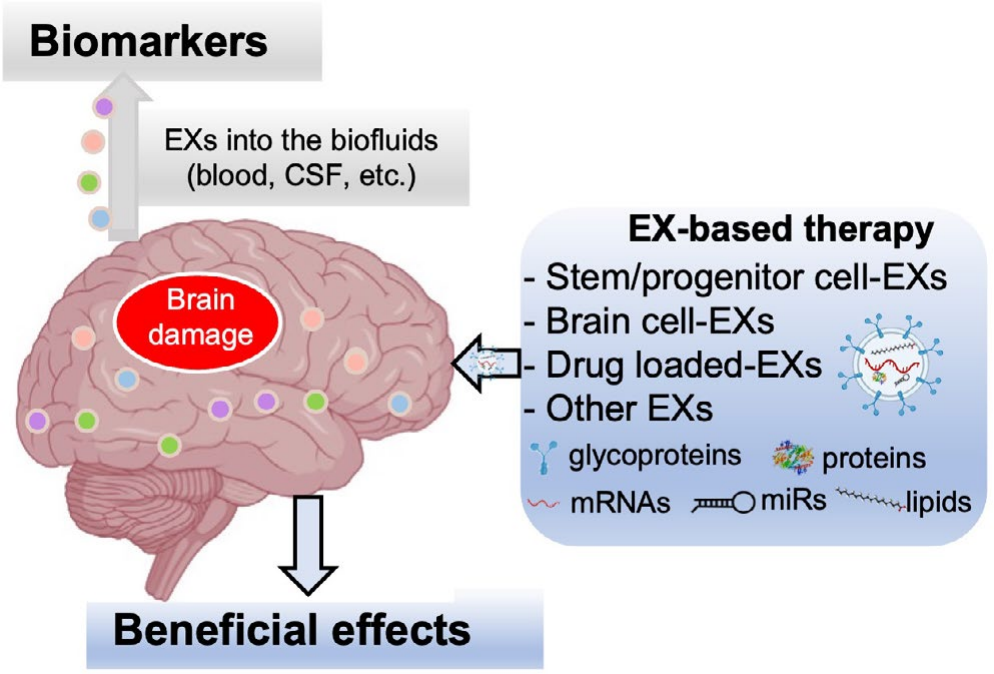
Exosomes are implicated in the central nervous system (CNS) diseases by mediating cell‐cell communications in the brain and modulating cellular functions through their carried molecules. Due to the nanosize and easy to cross blood‐brain barrier, exosomes hold great clinical application potentials for the CNS diseases by serving as biomarkers and therapeutic approaches

Although EXs display promising therapeutic effects on CNS diseases in preclinical studies, EX‐based therapy for treating CNS diseases in humans remains challenging. The production of engineering EXs mimics might overcome challenges related to sterility and mass production. For the safety and efficiency of EXs application in the clinic, there are still many challenges/concerns that need to be solved, such as selection of parent cells, EX isolation and purification, EX storage condition, optimal dose, and timing. To be used as a drug delivery system for targeting CNS diseases, besides to effectively isolate enough EXs, cellular and molecular contaminations must be cleared off. How to sufficiently load drugs, loading capacity, the targeting efficiency and standardize EX dosing, etc, are also waiting to be answered in future studies (Figure 2).

## CONFLICT OF INTEREST

None.

## Data Availability

The datasets generated during the current study are available from the corresponding author upon reasonable request.
